# Diseased Prize Cattle

**Published:** 1858-07

**Authors:** 


### THE EPITOME OF SANITARY LITERATURE.

Diseased Prize Cattle. The work of Mr. Gant † has excited very great attention in the agricultural world. The inquiry which he has instituted is, as he rightly claims, one of national interest and importance. He shows that the English method of feeding cattle is based on a vicious principle; that the present system of overfeeding leads to destruction of the muscular tissue, destroys the healthy action of the heart, and induces pathological changes, the recital of which would be sufficient to disgust the least delicate and fiercest appetite. With a boldness which does him immense credit, Mr. Gant, at the last Smithfield Cattle Show, followed up the labouring Prize Animals to their shambles, and after the butcher had performed the sacrificial rite, examined the bodies of these victims with a pathologist's knowledge. The diseased condition produced in these animals may be termed, says the writer, "conversion into fat, as expressive of the apparent change which has ensued; but on closer examination with the microscope, I would ascribe the change itself to the substitu-

---

† Evil Results of Overfeeding Cattle. A New Inquiry, illustrated with Coloured Engravings. By Frederick J. Gant. London: Churchill. 1858.

tion of fat (in the process of nutrition), for the proper structural elements—fibrillæ—of muscle, and not to the actual transformation of those elements into fat." He also adds,

"We should therefore expect in vain to replenish our own muscles by the use of such food, nor should animals thus over-fed be regarded as prize specimens of rearing and feeding. The heart, being converted into fat, no longer retains its contractile power, but beats feebly and irregularly. The blood, therefore, now moves onward in a slow and feeble current. Hence the panting breathlessness due to stagnation of blood in the lungs, which the heart labours (in vain) to remove, while the skin and extremities are cold. Hence the stupid, heavy-headed expression of a congested brain, and the blood-stained appearance of meat after death. The slightest exertion to an animal under such circumstances, might suddenly prove fatal. Were a man, in this condition, to present himself at an assurance office, it would refuse to insure his life at any premium. Yet, under similar circumstances, a sheep is awarded gold and silver medals, and its feeder a prize of £20."

